# Sexo pago e moral de nação: uma história da comercialização do sexo na Argentina do século XX

**DOI:** 10.1590/S0104-59702026000100042

**Published:** 2026-10-19

**Authors:** Marina Silva Duarte

**Affiliations:** i Professora, Departamento de História, Universidade Federal de Minas Gerais. Belo Horizonte – MG – Brasil. marinaduarte@ufmg.br

**Figure:**
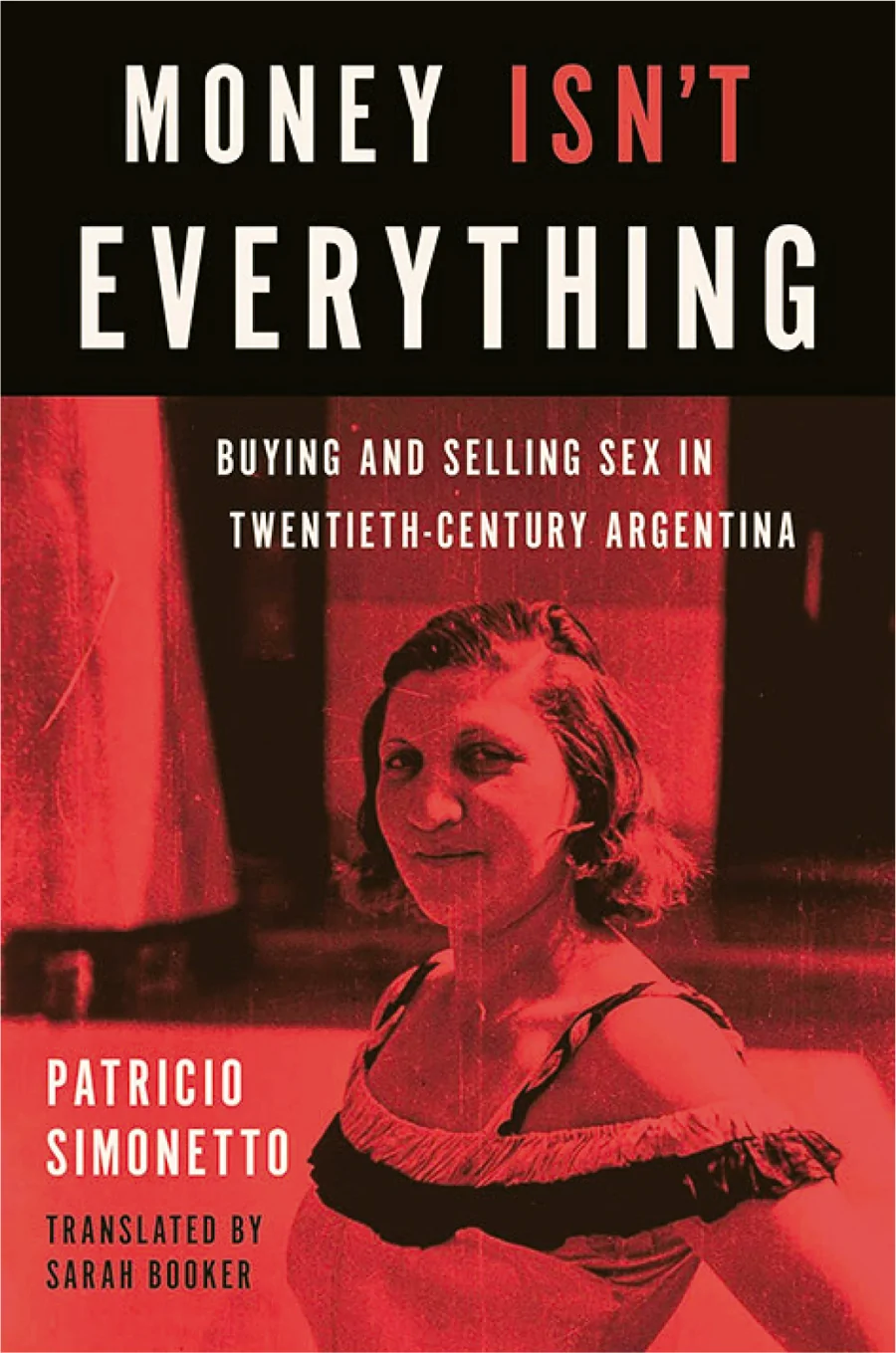

A tradução para o inglês (*Money isn’t everything: buying and selling sex in twentieth-century Argentina*) do livro de Patricio Simonetto, realizada por Sarah Booker, representa uma grande contribuição da pesquisa histórica para os estudos sobre sexualidade e gênero – especialmente em tempos de ataques contra esse campo do saber (Fassin, 2021). Simonetto propõe uma ambiciosa história social da comercialização do sexo na Argentina do século XX, entrelaçando práticas cotidianas, disputas morais e políticas nacionais de regulação do comércio sexual. O período estudado se estende desde a década de 1920, que antecede a promulgação da primeira lei abolicionista em 1936, até 1984, quando a proibição foi reafirmada e aplicada nacionalmente. Trata-se de um recorte extenso que cobre décadas de tensões entre políticas proibicionistas e práticas de tolerância, repressão seletiva, informalidade e negociações locais. O trabalho é exemplar na maneira como articula arquivos jurídicos, relatos da imprensa, correspondências e tratados médicos e policiais para construir uma narrativa multifacetada, em que o comércio do sexo aparece não apenas como uma prática econômica, mas como um campo de disputas simbólicas sobre gênero, raça, sexualidade e nação.

Simonetto faz uma análise refinada das fontes, que não são tratadas como espelhos da realidade, mas como arenas discursivas em que se cruzam interesses, coerções e estratégias. Seus arquivos incluem processos da prisão de Olmos, relatórios da Liga das Nações, correspondência entre militares e promotores de saúde, e até entrevistas com cafetões conduzidas por investigadores internacionais. Em vez de filtrar esses materiais por critérios de veracidade ou coerência, o autor os lê como traços de subjetividades em conflito e práticas sociais de negociação. Essa multiplicidade de tipos de fontes permite que Simonetto dê atenção tanto às formas institucionais de controle quanto às experiências dos indivíduos.

O maior mérito de seu estudo é articular as práticas de diferentes atores – das mulheres que vendem sexo aos compradores, negociadores, agentes da repressão, intelectuais, jornalistas e médicos – com o discurso jurídico e moral do Estado, que busca abolir o mercado do sexo por meio da legislação. Nesse sentido, o estudo revela como o Estado, por meio da interferência e repressão às relações sexuais tarifadas, estabelece um discurso moral que busca forjar a sexualidade aceita e almejada pela nação. O livro demonstra, então, de maneira muito detalhada e analítica, como foi construído um “nacionalismo sexual” (Mosse, 2020; Jaunait, 2014) na Argentina, cujo discurso moral e jurídico se baseava na proibição da venda de sexo. Ao longo dos capítulos, o autor demonstra como esse horizonte proibicionista – que se inicia com a Ley de Profilaxis 12.331, de 1936, a qual abole os bordéis e instaura um regime abolicionista – não se impôs sem resistências, sobretudo no que diz respeito ao entendimento de alguns militares e de figuras políticas importantes, que consideravam ser um direito dos homens o acesso ao sexo tarifado.

Esse jogo de escalas entre os sujeitos da história e as leis da nação está presente em todos os capítulos, que traçam uma trama das interações entre prostituição, moralidade pública, negociações locais e formas de regulação na Argentina do século XX. O primeiro capítulo, “The transnational circulation of pimps and prostitutes”, analisa os fluxos transatlânticos de cafetões e prostitutas, com destaque para os circuitos que conectavam a Europa, a Argentina e países do Cone Sul, como Brasil e Uruguai. O autor explora esse contexto que precede a era do proibicionismo e demonstra como a figura do proxeneta serviu para articular noções de raça e sexualidade (Dudink, 2013) em uma Argentina que buscava estabelecer barreiras à imigração. No segundo capítulo, “Soldiers, physicians, catholics, and unionists against the abolition of regulated prostitution”, Simonetto explora as reações de diferentes setores – militares, médicos, católicos e sindicalistas – à abolição do sistema de prostituição regulamentada. O capítulo destaca a articulação entre masculinidade, controle estatal e disputas em torno da sexualidade.

O terceiro e o quarto capítulos deslocam o foco para a mulher. “Selling sex in prison: women, punishment, and the judicial system” analisa a experiência de mulheres encarceradas por vender sexo entre 1936 e 1984, e discute os mecanismos de punição e controle moral que operavam na intersecção entre polícia, justiça e gênero. A partir das fontes, o autor enfatiza os laços e as relações que as mulheres construíam no sistema prisional. “Temporary work and paid sex in rural Buenos Aires” apresenta o impacto das dinâmicas do trabalho rural e da precariedade econômica na entrada de mulheres no mercado do sexo entre 1936 e 1960. Nesses dois capítulos, fica evidente que, mesmo quando se busca evidenciar a agência das mulheres, a estrutura do mercado do sexo entre homens clientes e mulheres prestadoras de serviço – foco do livro – constitui um sistema no qual a mulher, sobretudo a mais pobre, está submetida a constante violência.

O quinto capítulo, “The consumption of paid sex and worker culture”, analisa como o consumo de sexo pago era parte das práticas culturais de trabalhadores urbanos e rurais. A partir de depoimentos de clientes em processos penais contra supostos proxenetas, o autor mostra como os homens compreendiam esse consumo como um direito natural que reafirmava sua masculinidade. O pagamento por sexo, normalizado por rituais coletivos e discursos compartilhados, reforçava hierarquias de gênero e sexualidade. O último capítulo, “Police, prostitutes, and clients: a new order in the sexual market”, investiga as mudanças nas dinâmicas do mercado sexual no norte e no oeste da província de Buenos Aires entre 1960 e 1980. Nesse período, é observado o surgimento de circuitos informais de compra e venda de sexo, especialmente em zonas rurais e ao longo das estradas. A análise destaca como reformas legais e campanhas de moralização pública alteraram as formas de regulação do sexo pago, reorganizando as relações entre prostitutas, clientes e a polícia.

O livro expõe o mosaico complexo da realidade do sexo tarifado na Argentina. Simonetto rejeita explicações morais, deterministas ou dicotômicas – como vítima *versus* escolha, trabalho livre *versus* escravidão sexual – e propõe uma leitura histórica das práticas sexuais pagas que reconhece sua instabilidade, ambiguidade e complexidade. Apesar do reconhecimento dessa complexidade, minha leitura é de que o livro evidencia como o sexo tarifado entre homens e mulheres constituiu um sistema que forja a sexualidade masculina como dominante e, ao mesmo tempo, sujeita o corpo feminino, principalmente das mulheres pobres. Trata-se de uma excelente contribuição para o campo dos estudos históricos sobre a história da prostituição, que vem, nas últimas décadas, ampliando suas possibilidades de pesquisa.

## Data Availability

Não estão em repositório.
